# An Optimized Transient Dual Luciferase Assay for Quantifying MicroRNA Directed Repression of Targeted Sequences

**DOI:** 10.3389/fpls.2017.01631

**Published:** 2017-09-20

**Authors:** Richard L. Moyle, Lilia C. Carvalhais, Lara-Simone Pretorius, Ekaterina Nowak, Gayathery Subramaniam, Jessica Dalton-Morgan, Peer M. Schenk

**Affiliations:** Nexgen Plants Pty Ltd., School of Agriculture and Food Sciences, University of Queensland, Brisbane QLD, Australia

**Keywords:** microRNA prediction, target recognition, dual luciferase assay, miR529b, OsSPL14, SBP-box family genes

## Abstract

Studies investigating the action of small RNAs on computationally predicted target genes require some form of experimental validation. Classical molecular methods of validating microRNA action on target genes are laborious, while approaches that tag predicted target sequences to qualitative reporter genes encounter technical limitations. The aim of this study was to address the challenge of experimentally validating large numbers of computationally predicted microRNA-target transcript interactions using an optimized, quantitative, cost-effective, and scalable approach. The presented method combines transient expression via agroinfiltration of *Nicotiana benthamiana* leaves with a quantitative dual luciferase reporter system, where firefly luciferase is used to report the microRNA-target sequence interaction and Renilla luciferase is used as an internal standard to normalize expression between replicates. We report the appropriate concentration of *N. benthamiana* leaf extracts and dilution factor to apply in order to avoid inhibition of firefly LUC activity. Furthermore, the optimal ratio of microRNA precursor expression construct to reporter construct and duration of the incubation period post-agroinfiltration were determined. The optimized dual luciferase assay provides an efficient, repeatable and scalable method to validate and quantify microRNA action on predicted target sequences. The optimized assay was used to validate five predicted targets of rice microRNA miR529b, with as few as six technical replicates. The assay can be extended to assess other small RNA-target sequence interactions, including assessing the functionality of an artificial miRNA or an RNAi construct on a targeted sequence.

## Introduction

The discovery of regulatory RNA has contributed to a paradigm shift in the field of molecular biology (Morris and Mattick, 2014). Previously, the central dogma stated that the primary role of RNA was to convert the DNA code for protein synthesis, via messenger RNA and the action of non-coding ribosomal RNAs and transfer RNAs. It is now known that various additional classes of non-coding RNAs play complex and elaborate roles in the regulation of genome organization and gene expression. Small interfering RNAs (siRNAs) and microRNAs (miRNAs) are central to this field of study, and these small RNA (sRNA) classes have been shown to regulate a wide range of physiological, developmental, stress, and defense processes in plants (Mallory and Vaucheret, 2006; Sunkar et al., 2007; Chen, 2009, 2012; Chuck et al., 2009; Baulcombe, 2015).

Briefly, siRNAs are ∼21–24 nucleotide (nt) molecules generated from double-stranded RNA precursors. siRNAs act on RNA molecules by initiating canonical RNA degradation, or upon DNA molecules via RNA-directed DNA methylation (Chen, 2009). siRNAs may originate from a variety of sources including repetitive sequences, transposons, centromeres, natural sense–antisense transcripts, hairpin RNAs, trans-acting siRNA generating transcripts, and pathogens such as plant viruses (Smith et al., 2011; Axtell, 2013; Borges and Martienssen, 2015). The miRNA class of small RNAs are ∼21 nt and are processed from non-coding RNA precursor transcripts that form a stem–loop structure (Axtell et al., 2011). When complexed with Argonaute proteins, miRNAs function as negative regulators by recognizing partially or perfectly complementary transcript target sequences and triggering a cleavage event or translational repression.

Advances in sequencing technology have facilitated the discovery of vast populations of sRNAs, many of which are developmentally regulated (Zabala et al., 2012; Sternes and Moyle, 2014). Similarly, deep sequencing has led to large collections of transcriptome datasets that can be computationally mined for transcript sequences that are predicted to be targeted by sRNAs. For example, the psRNAtarget resource allows users to screen preloaded or user defined transcriptomes for transcripts with complementarity to a sRNA of interest (Dai and Zhao, 2011). As a consequence, large numbers of computationally predicted targets of sRNAs have been reported. The challenge remains how to experimentally validate large numbers of computationally predicted sRNA-target transcript interactions via the most accurate, cost-effective, and least labor intensive or time consuming approach possible.

The functions of miRNA have previously been validated via analysis of phenotypes in stably transformed transgenic and mutant plant lines (Palatnik et al., 2003; Mallory et al., 2004). Stable transformation of a reporter gene construct containing a miRNA target sequence has also been used to study developmental miRNA expression patterns in plants (Moyle et al., 2014). However, these approaches are time consuming, laborious, and are not practical for high throughput testing. Detection of miRNA-directed cleavage of targeted transcripts has been achieved using *in vitro* methodologies such as 5′ RACE (Llave et al., 2002; Sternes and Moyle, 2014), but these approaches will not detect miRNA-directed translational inhibition and can therefore produce false negative results. Transient assay systems, including the *Agrobacterium tumefaciens* infiltration assay (agroinfiltration) on *Nicotiana benthamiana* leaves, and subsequent molecular analyses have been used to study miRNA-target gene interactions (Llave et al., 2002). Tagging reporter genes with target sequences and co-agroinfiltration of these constructs with a corresponding miRNA precursor construct has also been used to study miRNA-target interactions (Franco-Zorrilla et al., 2007). The use of green fluorescent protein as the reporter provides a visual indicator of target sequence repression (Franco-Zorrilla et al., 2007). However, this qualitative assay is not suitable for detecting subtle target repression effects or requires further molecular analysis to quantitate any miRNA-directed repression of the reporter gene expression.

Recently the dual luciferase (LUC) assay was applied to study complementarity requirements for miRNA-target interactions using the transient agroinfiltration assay on *N. benthamiana* leaves (Liu et al., 2014; Liu and Axtell, 2015). In the dual LUC assay, the Renilla LUC expression cassette acts as an internal control to standardize expression between replicates, while the firefly LUC expression cassette containing the predicted target sequence is used to report miRNA interaction. The light emitted by the LUC reporters during the assay can be measured in a luminometer and this quantitative benefit provides an important advantage over other qualitative reporter systems. Multiple miRNA and target sequence pairings can be tested simultaneously, potentially providing a relatively quick way to screen large numbers of predicted miRNA-target sequence interactions. However, another recent report found that the firefly LUC reporter was inhibited by plant extracts and that considerable dilution was necessary to eliminate the inhibitory effect and generate accurate LUC readings with lower variability (Moyle and Birch, 2013). Furthermore, analyses of parameters such as the optimal ratio of miRNA precursor construct to target reporter construct or incubation period post-agroinfiltration have not been reported.

This study investigates key parameters and experimental conditions required to maximize miRNA-directed repression of a target sequence when using a transient assay that combines co-agroinfiltration of *N. benthamiana* leaves with the quantitative dual LUC assay system. We report the minimum leaf extract dilution required to avoid inhibition of the firefly LUC reporter. We also report the optimal ratio of miRNA precursor construct to reporter construct and the minimum incubation period post-agroinfiltration to maximize miRNA directed knockdown of target gene expression. Additionally, we describe improved expression of the Renilla LUC reporter under the control of a tomato *ACTIN* promoter, compared to the strongly wound induced but relatively weak expressing *A. tumefaciens NOS* promoter. We applied the assay to validate computationally predicted targets of rice miRNA529b with just six replicate samples, demonstrating the scalability of the optimized assay conditions. Considerations for experimental design, current limitations, and extended applications of the assay are discussed.

## Materials and Methods

### Constructs and Plasmids

**FIGURE 1:**
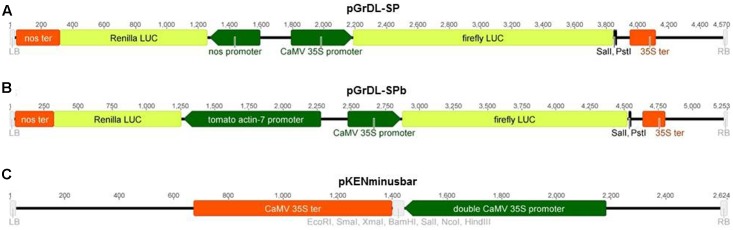
Schematic of the T-DNA regions of constructs used in the study. **(A)** Dual LUC assay plasmid pGrDL_SP, with *Sal*I and *Pst*I restriction sites for target sequence insertion. **(B)** Dual LUC assay plasmid pGrDL_SPb, with *Sal*I and *Pst*I restriction sites for target sequence insertion. **(C)** Expression plasmid pKENminusbar with multiple cloning sites for miRNA precursor insertion.

The plasmids used in this study were constructed by modifying the pGreen dualLUC 3′ UTR sensor plasmid described by Liu et al. (2014). The pGreen dualLUC 3′ UTR sensor plasmid was first digested with *Sal*I restriction enzyme (New England Biolabs) followed by T4 DNA polymerase (New England Biolabs) treatment and self-ligation, in order to inactivate the *Sal*I restriction site between the *NOS* promoter and the Renilla LUC coding sequence (verified by Sanger sequencing of the *NOS* promoter-Renilla LUC coding sequence junction). The resulting intermediary plasmid was then double digested with *Eco*RI and *Pst*I (New England Biolabs), followed by Antarctic Phosphatase treatment (New England Biolabs). An adaptor containing a *Sal*I restriction site was made by annealing two complementary primers with 5′ phosphate additions and *Eco*RI and *Pst*I overhangs. The adaptor was subsequently ligated into the intermediary plasmid to construct pGrDL_SP (**Figure 1A**).

The *NOS* promoter in pGrDL_SP was replaced with a tomato *ACTIN* promoter to construct plasmid pGrDL_SPb (**Figure 1B**). This was achieved via PCR amplification of the tomato *ACTIN-7* promoter (NCBI reference sequence NM_001308447.1, and phytozome genomic region SL2.50ch03:50833930…50834937) using a 5′ primer containing an *Fsp*I restriction site and a 3′ primer containing a *Sal*I restriction site (primers SlActProm_F_FspI and SlActProm_R_SalI, Supplementary Table S1). The amplicon was digested with *Fsp*I and *Sal*I to create compatible overhangs for cloning into the *Fsp*I and *Psp*XI restriction sites of pGrDL_SP. The replacement of the *NOS* promoter with the tomato *ACTIN* promoter to form plasmid pGrDL_SPb was verified by Sanger sequencing. The sequences of pGrDL_SP and pGrDL_SPb have been deposited into NCBI GenBank with accession numbers KX758647 and KX758648, respectively. The pGrDL_SP and pGrDL_SPb plasmids have been submitted to the Addgene plasmid repository, with plasmid identification numbers 83204 and 83205 respectively^1^.

Target site adaptors were made by annealing two complementary primers (Supplementary Table S1), synthesized with 5′ phosphate additions and *Sal*I and *Pst*I overhangs (synthesized by Integrated DNA Technologies). Approximately 10 nmol of each primer were mixed together in a volume of 60 μL of DNAse-free water, heated to ∼90°C for 5 min in a water bath and allowed to slowly cool to room temperature (at a rate of ∼1 degree per minute). The resulting adaptors were diluted 50-fold prior to directional cloning into a *Sal*I and *Pst*I digested and Antarctic Phosphatase (New England Biolabs) treated pGrDL_SPb plasmid, and subsequently verified by Sanger sequencing (Australian Genome Research Facility).

The rice miRNA precursor for miR529b (accession MI0005804 from www.mirbase.org) was synthesized with flanking restriction enzyme sites (GenScript) and cloned between the double *Cauliflower mosaic virus* (*CaMV*) 35S promoter and 35S terminator of the expression plasmid pKENminusbar (**Figure 1C**). A similar sized construct, derived from *Capsicum chlorosis virus*, was cloned into pKENminusbar and used as a control. This control sequence (Supplementary Table S3) is predicted to form a secondary structure similar to a miRNA precursor, but does not contain sufficient homology to produce small RNA capable of interacting with the reporter gene target sequence.

### Agroinfiltration of *Nicotiana benthamiana* Leaves

Expression plasmids were transformed by electroporation into *A. tumefaciens* strain GV3101 (harboring pSOUP) and plated on LB agar media with rifampicin (25 μg/mL), kanamycin (50 μg/mL), and tetracycline (10 μg/mL) selection. Starter cultures were prepared by inoculating a single colony in 2 mL of LB with rifampicin, kanamycin and tetracycline selection, and grown overnight at 28°C in a shaking incubator. Starter culture was used to inoculate the working culture (typically 20–50 mL), which was grown overnight under the same media, selection and incubation conditions. Working cultures were harvested by centrifugation at 24°C and 2,400 × *g* for 15 min. Cell pellets were resuspended in 10 mM MgCl_2_ and the OD_600_ adjusted to 0.5 prior to the addition of acetosyringone (200 μM). The resulting cell cultures were stored in the dark at room temperature for either 4 or 24 h prior to infiltration.

*Nicotiana benthamiana* plants were grown at room temperature and 16 h light per day, under LumiBar LED strip lighting (LumiGrow) intensity setting four. Seedlings were grown for approximately 3–4 weeks prior to agroinfiltration. Three expanded leaves per plant were infiltrated by applying pressure on the abaxial surface of the leaf with a disposable 5 mL syringe containing the *Agrobacterium* suspension. Each leaf was treated as a biological replicate. Agroinfiltrated plants were incubated for a specified period (typically 72 h) in a growth chamber set to 22°C with 16 h light per day. Agroinfiltrated leaves were harvested individually, snap-frozen in liquid nitrogen, powdered using a ball mill tissue-lyser (Retsch), and stored at -80°C prior to measurement in dual LUC assays.

### Dual Luciferase Assay

Dual luciferase (LUC) assay extracts were prepared using the Dual Luciferase Reporter Assay System kit (Promega). Typically ∼5 mg of powdered tissue was mixed with 100 μL of the passive lysis buffer (PLB) provided in the Dual Luciferase Reporter Assay System kit, and the cellular debris pelleted by centrifugation at 7,500 × *g* for 1 min. The supernatant was typically diluted 20-fold in PLB and 15 μL loaded into a well of a white flat bottom Costar 96 well plate (Corning). The assay was performed using a GloMax 96 microplate luminometer (Promega). The dual injectors were used to introduce 75 μL of luciferase assay reagent and Stop & Glo reagent, respectively, per well. Luciferase assay reagent and Stop & Glo reagent components are provided in the Dual Luciferase Reporter Assay System kit (Promega).

Statistical analysis of the resulting data was performed using GraphPad Prism 6 software.

## Results

### The Firefly Luciferase Reporter Is Inhibited by Concentrated *N. benthamiana* Leaf Extracts

**FIGURE 2:**
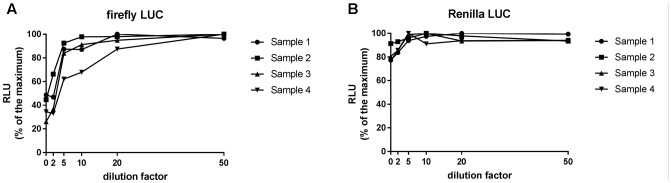
Relative luminescence units emitted by firefly LUC **(A)** and Renilla LUC **(B)** activity across a range of dilution factors applied to *Nicotiana benthamiana* leaf extracts. Firefly LUC activity is inhibited by concentrated *N. benthamiana* leaf extracts. 20-fold dilution of a 5 mg per 100 μL extract is required to diminish the inhibitory effect.

Previous studies in sugarcane revealed that undiluted plant extracts can inhibit firefly luciferase activity (Moyle and Birch, 2013). We first investigated if agroinfiltrated *N. benthamiana* leaf extracts were capable of inhibiting firefly or Renilla LUC activity and what dilution factor, if any, is required to negate the inhibition of activity. Extracts were prepared from *N. benthamiana* leaves agroinfiltrated with the dual LUC plasmid pGrDL_SPb (**Figure 1B**) at a starting concentration of 5 mg per 100 μL of PLB. LUC activity measurements revealed substantial inhibition of firefly LUC activity in undiluted extracts (**Figure 2**). The level of inhibition varied substantially from sample to sample, with LUC activity levels between 26 and 49% of that in diluted samples. In contrast, inhibition of Renilla LUC activity was minimal in the undiluted leaf extracts (**Figure 2**). The inhibition effect on firefly LUC was lessened by dilution, plateauing out after 20-fold dilution (**Figure 2**). Thus we recommend preparing at least 20-fold dilutions of extracts made from no more than 5 mg leaf tissue per 100 μL PLB to negate any inhibitory impact on firefly LUC activity. All subsequent optimisation experiments presented in this article utilized 20-fold dilution of extracts containing < 5 mg leaf tissue per 100 μL PLB.

### The Tomato *ACTIN* Promoter Drives Higher Levels of Renilla LUC Expression than the Wound-Inducible *NOS* Promoter

The pGreen dualLUC 3′ UTR sensor plasmid described by Liu et al. (2014) was modified to allow directional cloning of target sequences in the firefly LUC 3′ UTR using *Sal*I and *Pst*I restriction sites. This modified plasmid was named pGrDL_SP (for pGreen dual LUC *Sal*I and *Pst*I restriction sites) (**Figure 1A**). The Renilla LUC expression cassette in pGrDL_SP is driven by the *NOS* promoter. It is reported that *NOS* promoter activity can be strongly induced by wounding in leaf tissues (An et al., 1990). As agroinfiltration causes a degree of wounding to the leaf tissue, we hypothesized that the wound-induced expression may contribute to variability. Furthermore, the Renilla LUC expression cassette under the control of the *NOS* promoter yields an order of magnitude fewer relative light units (RLUs) than the firefly LUC expression cassette under the control of the *CaMV* 35S promoter. To address these concerns, we substituted the *NOS* promoter with a tomato *ACTIN* promoter sequence to form plasmid pGrDL_SPb (**Figure 1B**). We then compared the RLU yield from the Renilla LUC reporter generated by each plasmid after agroinfiltration of *N. benthamiana* leaves.

**FIGURE 3:**
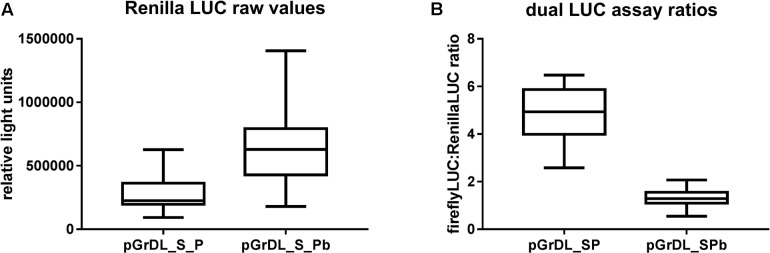
Comparison of dual LUC plasmids pGrDL_SP and pGrDL_SPb. **(A)** Raw Renilla LUC relative light units generated from each plasmid. **(B)** Dual LUC ratios obtain from each plasmid. The results are depicted as box and whisker plots, with interquartile range boxes and min/max whiskers (*n* = 18).

The tomato *ACTIN* promoter drove substantially higher Renilla LUC expression on average than the *NOS* promoter, thereby increasing the sensitivity of the assay (**Figure 3A**). Accordingly, the RLU values generated from the tomato *ACTIN*-Renilla LUC expression cassette were closer to those obtained from the *CaMV* 35S-firefly LUC expression cassette, resulting in lower dual LUC ratios generated from pGrDL_SPb (**Figure 3B**). The reduced interquartile range of the dual LUC ratios generated from pGrDL_SPb indicates the replacement of the *NOS* promoter with the tomato *ACTIN* promoter may have also reduced variability. The dual LUC plasmid pGrDL_SPb was used in all further optimisation experiments.

### An Optimal Ratio of Precursor Construct to Reporter-Target Construct Is Required to Maximize the miRNA-Directed Repression of Target Gene Expression

The miRNA precursor plasmid used in this study was constructed by cloning the rice miR529b precursor sequence under the control of the double *CaMV* 35S promoter in expression plasmid pKENminusbar (**Figure 1C**). Rice miR529b is evolutionarily related to miR156 (Zhang et al., 2015; Morea et al., 2016). While miR156 is highly conserved in land plants, the rice miR529b sequence was selected for optimisation of the assay because the miR529b miRNA is not endogenous to *N. benthamiana*, and therefore the results are not likely to be confounded by endogenous miRNA interaction with the target sequence. A perfectly matching (PM) complementary sequence to rice miR529b was selected as the target sequence and cloned into the 3′ UTR of the firefly LUC construct within the dual LUC reporter plasmid pGrDL_SPb. Although the 3′ and central seed regions of miR156 overlap the targeted sites of miR529b, it is unlikely endogenous miR156 could effectively target the cloned miR529b target sequence due to the absence of target sequence complementary to the important 5′ seed region (Liu et al., 2014).

**FIGURE 4:**
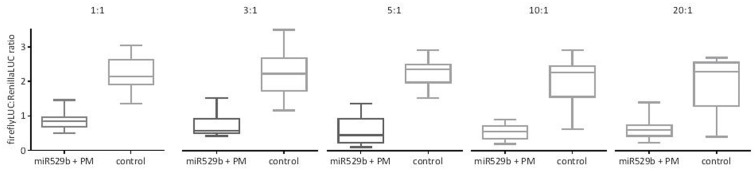
miR529b-mediated repression of perfectly matching (PM) target reporter gene expression in transient co-agroinfiltration assays with different precursor culture to target culture ratios. The controls consist of assays where the miR529b precursor culture was replaced with a precursor construct culture that does not target the miR529b PM sequence. The results are depicted as box and whisker plots, with interquartile range boxes and min/max whiskers (*n* = 12).

A previous study used a 1:1 ratio of miRNA precursor plasmid *Agrobacterium* culture and dual LUC plasmid *Agrobacterium* culture (Liu et al., 2014; Liu and Axtell, 2015). To investigate the optimal ratio of miRNA precursor to dual LUC plasmid cultures to maximize any miRNA-mediated repression, co-agroinfiltration was undertaken with varying ratios of rice miRNA529b precursor culture to PM target in dual LUC reporter pGrDL_SPb culture. While knockdown of expression was evident at a 1:1 ratio (2.5-fold knockdown on average), increasing the proportion of miR529b precursor culture to a 5:1 or 10:1 ratio caused the greatest knockdown of expression (between 3.8 and 5.2-fold on average) of the firefly LUC reporter construct containing the target sequence (**Figure 4**). As a consequence, all further optimisation steps presented in this article used a 10:1 ratio of miRNA precursor culture to target in dual LUC reporter pGrDL_SPb culture.

### At Least Three Days Incubation Post-agroinfiltration Are Required to Maximize miRNA-Directed Knock-Down of Target Gene Expression

**FIGURE 5:**
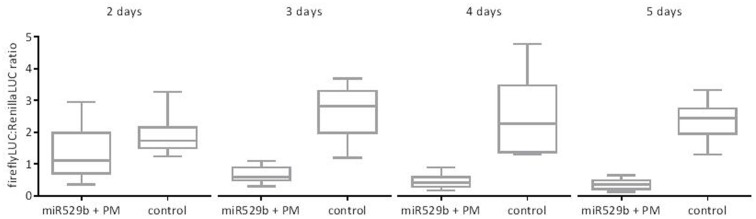
Dual LUC assays reporting miR529b-mediated repression of a PM target within the firefly reporter gene construct, after a range of incubation periods post co-agroinfiltration. The controls consist of assays where the miR529b precursor construct culture was replaced with a construct that is predicted to form a secondary structure but does not target the mir529b PM sequence. The results are depicted as box and whisker plots, with interquartile range boxes and min/max whiskers (*n* = 12).

To assess the optimum incubation period for miRNA-directed knockdown of reporter containing the target sequence, agroinfiltrated leaves were harvested each day for 5 days post-infiltration. The expression of the Renilla LUC reporter was near background at day 1, and therefore no data was analyzed at that time point. Both the firefly LUC and Renilla LUC raw values were relatively low at 2 days post-agroinfiltration, and the dual LUC assay ratio indicated a minor knock-down effect that did not pass an unpaired *t*-test (*P* = 0.051, *n* = 12). A strong and highly significant knock-down of expression (*P* < 0.0001, *n* = 12) was detected 3 days post-agroinfiltration (**Figure 5**). A similar if not greater knockdown of expression was found at 4 and 5 days post-agroinfiltration. However, the raw RLU values peaked at day 3 and declined from day 4 to day 5. Therefore we recommend an incubation period of 3 days post-agroinfiltration.

### Comparison of Incubation Periods of 4 h vs. 24 h Post-addition of Acetosyringone

**FIGURE 6:**
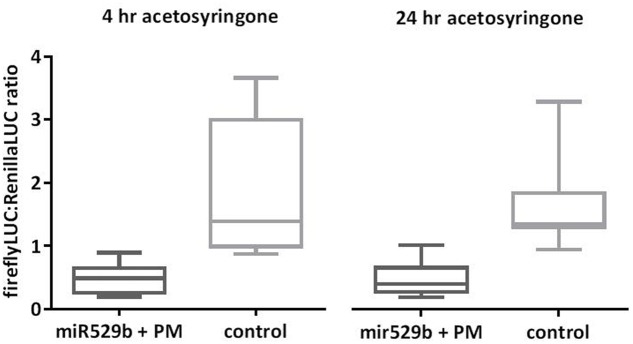
Comparison of 4 h versus 24 h acetosyringone incubation periods. Increasing the incubation period of *Agrobacterium* cultures from 4 to 24 h post-addition of acetosyringone did not enhance the mi529b-directed knockdown of reporter-target expression. The controls consist of assays where the miR529b precursor culture was replaced with a precursor construct culture that does not target the miR529b PM sequence. Error bars represent SDs (*n* = 12).

Agroinfiltration protocols specify addition of acetosyringone to the *Agrobacterium* culture in order to induce the virulence genes. However, reported studies vary in the duration of this incubation period, ranging from 1 to 24 h (Koia et al., 2013; Liu and Axtell, 2015; Condori et al., 2016). To assess if longer incubation enhances miRNA-directed knock-down of targeted reporter gene expression, we compared 4 and 24 h incubation periods post-addition of acetosyringone to the *Agrobacterium* culture. Our results indicate no advantage in incubating *Agrobacterium* cultures for 24 h over 4 h post-addition of acetosyringone (**Figure 6**).

### Direct Comparison of the Optimized Dual LUC Assay with the Previously Reported Assay

**FIGURE 7:**
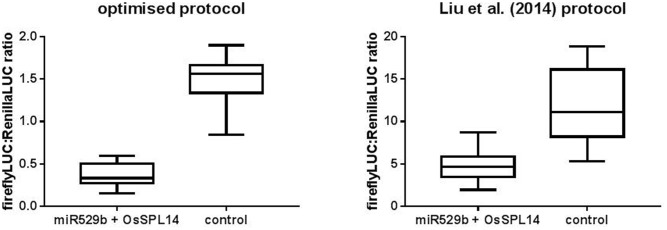
Comparison of the optimized dual LUC assay versus the prior art described by Liu et al. (2014). The optimized assay conditions include using pGrDL_SPb for *OsSPL14* target sequence cloning, a 10:1 ratio of rice miR529b precursor culture to target plasmid culture, 3 days incubation post-agroinfiltration of *N. benthamiana* leaves, and dual LUC assays on extracts diluted 20-fold. The optimized assay control consists of assays where the miR529b precursor culture was replaced with a precursor construct culture that does not target *OsSPL14* sequence. The Liu et al. (2014) assay conditions include using pGreen dualLUC 3′ UTR sensor plasmid for *OsSPL14* target sequence cloning, a 1:1 ratio of rice miR529b precursor culture to target plasmid culture, 2 days incubation post-agroinfiltration of *N. benthamiana* leaves, and dual LUC assays on undiluted extracts. The Liu et al. (2014) control consists of assays where the *OsSPL14* target plasmid culture was replaced with a plasmid culture that is not targeted by rice mir529b. The results are depicted as box and whisker plots, with interquartile range boxes and min/max whiskers (*n* = 12).

A previously published study used a dual LUC assay to quantify miRNA-mediated repression of targeted gene sequences (Liu et al., 2014). Following that study, a book chapter was published providing more detailed methodology (Liu and Axtell, 2015). A number of key differences exist between the prior method of Liu et al. (2014) and the optimized conditions presented in this study. Liu et al. (2014) used the wound inducible and relatively weak *NOS* promoter to drive Renilla LUC expression, whereas this study used the relatively strong tomato *ACTIN* promoter. Further, Liu et al. (2014) used a 1:1 ratio of miRNA precursor *Agrobacterium* culture to target plasmid *Agrobacterium* culture, incubated their plants for just 2 days post-agroinfiltration, and did not apply a dilution factor to their plant extracts prior to running the dual LUC assay. We hypothesized the optimisation described in this study would reduce variability and enhance the level of miRNA-mediated repression of target gene sequence constructs. A direct comparison of the two methods was undertaken pairing rice miR529b precursor with the known target sequence from *OsSPL14* cloned into either pGrDL_SPb or the pGreen dualLUC 3′ UTR sensor plasmid described by Liu et al. (2014). The optimized assay detected a greater knockdown of target gene expression (fourfold) than the previously described method (2.5-fold) (**Figure 7**). Importantly, variability was lower in the optimized assay as evidenced by the reduced inter-quartile range (**Figure 7**).

### Experimental Validation of Computationally Predicted Targets of Rice miR529b

**FIGURE 8:**
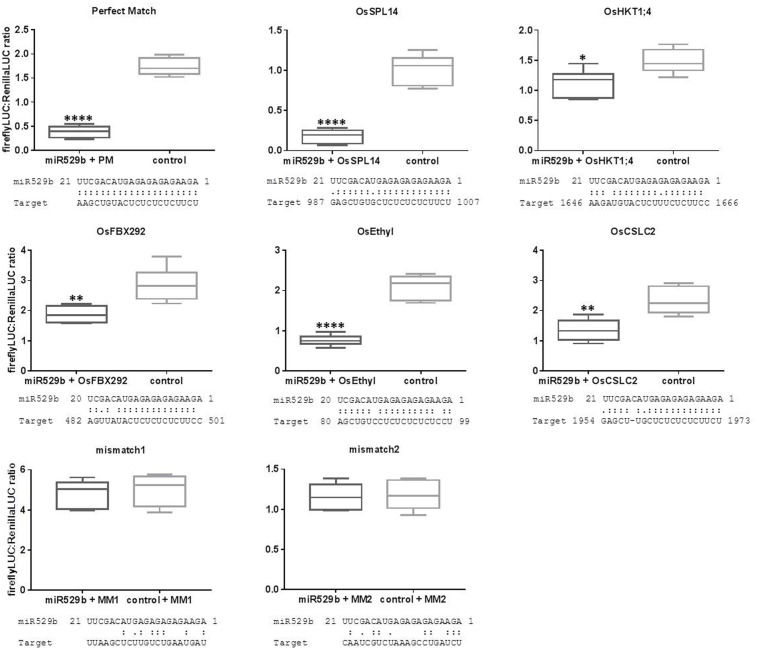
Experimental validation of rice transcript sequences computationally predicted to be targeted by rice miR529b. Each control consists of assays where the miR529b precursor culture was replaced with a precursor construct culture that does not target the rice sequences. Mismatch1 (MM1) and mismatch2 (MM2) negative control assays use target sequences with little complementarity to the miR529b miRNA. The results are depicted as box and whisker plots, with interquartile range boxes and min/max whiskers (*n* = 6). Asterisk represents statistically significant differences using a simple two-tailed unpaired *t*-test with ^∗^*P* < 0.05, ^∗∗^*P* < 0.005, and ^∗∗∗∗^*P* < 0.0001.

The optimized assay conditions of 10:1 miR529b precursor to reporter-target expression cultures including 3 days incubation post co-infiltration of *N. benthamiana* leaves and 20-fold dilutions of ∼5 mg/100 μL leaf extracts were used to experimentally validate computationally predicted targets. The psRNAtarget online resource was used to screen for rice transcript sequences that are computationally predicted targets of rice miR529b (Dai and Zhao, 2011). Five of the highest ranking predicted target sequences were cloned into the pGrDL_SPb reporter plasmid (the target sequence pairings and transcript identifiers are listed in Supplementary Table S2). The subsequent dual LUC assays revealed repression of the firefly LUC reporter when each of the five predicted target sequences were present in the construct (**Figure 8**). The strength of the repression effect varied between the different target constructs. The highest scoring predicted target sequence was from transcript *OsSPL14* and this target construct generated the strongest repression effect. Lower scoring predictions, including target sequences from transcripts *OsHXT1;4* and *OsFBX292*, produced relatively subtle repression of reporter expression. Although the sample size for each treatment was small (*n* = 6), the repression effect in each test case was significantly different to the control treatments using a simple unpaired *t*-test. Two target sequences (named mismatch1 and mismatch2) containing little complementarity to miR529b were also tested. No difference in expression level was observed when these control target sequences were co-infiltrated with the miR529b precursor or the control precursor, indicating the miR529b-dependent repression of predicted target sequences was not due to a secondary effect.

We note there were differences in the dual LUC ratios generated from each test construct when co-infiltrated with the control precursor construct. For example, the perfect match target construct generated average dual LUC ratios of 1.735 (± 0.07547 SEM, *n* = 6), while the *OsSPL14* and *OsFBX292* target constructs generated average dual LUC ratios of 1.013 (± 0.07543 SEM, *n* = 6) and 2.870 (± 0.2334 SEM, *n* = 6), respectively, in the control treatments. These constructs only vary by the sequence composition of the short target sequence inserted into the 3′ UTR of the firefly expression construct, yet these differences are sufficient to cause noticeable differences in the stability of the reporter gene expression in the controls. This variability between different test constructs highlights the need for appropriate controls. Previous reports have compared test constructs to a control construct containing a non-targeted sequence, assuming the stability of reporter expression is not affected by the different target sequences (Liu et al., 2014). However, our results suggest that different target sequences can influence the stability of reporter expression, compromising the integrity of results obtained using that control strategy, as any reduction in the dual LUC ratio may not be due to miRNA-target interaction but due to differing reporter transcript stability. As such, we suggest pairing each test construct with a non-functional precursor construct for a more robust control.

## Discussion

The quantitative nature of the firefly and Renilla LUC reporters provide a key advantage over most other qualitative reporter systems. A previous study showed that agroinfiltration paired with a dual LUC reporter assay can be used to quantify miRNA-mediated repression of target sequences (Liu et al., 2014). However, relatively large numbers of replicates were required and the variability of the assay was perhaps larger than expected, considering the presence of the Renilla LUC expression cassette as an internal control to standardize expression between replicates. This study aimed to optimize various parameters to increase the fidelity of the assay and reduce variability.

One important consideration comes from the little known discovery that plant tissue extracts can inhibit the activity of the firefly LUC enzyme. A study using firefly LUC to report promoter activity in transgenic sugarcane revealed that different plant tissue types inhibit firefly LUC activity to varying degrees (Moyle and Birch, 2013). The inhibiting effect was negated by dilution and the sensitive nature of the firefly LUC reporter system allows for a substantial degree of dilution before reporter measurements reach background values. No inhibition of Renilla LUC activity was found during a study utilizing the dual LUC assay in sugarcane (Chou and Moyle, 2014). Similarly, our results show that *N. benthamiana* leaf extracts can also inhibit firefly LUC activity, and that the inhibitory effect can be diluted out. A previously reported dual LUC assay method did not dilute *N. benthamiana* leaf extracts prior to undertaking dual LUC assay measurements to quantify miRNA-mediated target repression (Liu et al., 2014; Liu and Axtell, 2015). They did however, note a drop in firefly activity levels from samples in wells measured toward the end of a luminometer plate run, compared to samples position in wells measured earlier by the plate reader luminometer (Liu and Axtell, 2015). It was hypothesized that protease activity may have been responsible, although the inclusion of protease inhibitors had no impact (Liu and Axtell, 2015). We speculate that proteases are not responsible but rather one or more inhibitory compounds, perhaps phenolic compounds, are responsible for the observed effect. Fortunately the inhibition of firefly LUC activity can be relieved by dilution, and this strategy is feasible due to the sensitivity of the dual LUC assay. We believe appropriate dilution of the plant extract is likely the most important factor in reducing variability of the assay.

Other factors such as the increasing the ratio of miRNA precursor construct culture to target-reporter construct culture, and increasing the plant incubation period post-agroinfiltration provided incremental improvements in the sensitivity of the assay when using miR529b as the precursor miRNA. It is possible other miRNA precursors may require individual optimisation to find the optimal ratio for reporting knockdown of target gene expression. It is likely that this approach could be applied to other plant systems that are amenable to agroinfiltration. Alternatively, biolistic or PEG-mediated DNA transfer methods could be used to co-introduce the precursor and reporter-target constructs into a wider range of plant cells, tissues and host plants.

We have demonstrated this optimized transient co-agroinfiltration dual LUC assay can be used to quickly validate multiple computationally predicted targets of rice mir529b on as few as six leaves using *N. benthamiana* as the host plant. The PM target sequence and the most complementary predicted rice transcript target sequence from *OsSPL14*, a SBP-box gene family member, produced the largest miRNA-mediated repression effect. The predicted rice mir529b – *OsSPL14* target sequence interaction produced the lowest E value of just 0.5; the only mismatches being wobble G:U pairings 3′ of the important 5′ and central seed regions of the miRNA. The four remaining target sequences tested had predicted miRNA interaction E values of 2.5, with up to three mismatches at various positions across the rice miR529b. Each predicted interaction caused a miRNA-directed repression effect, although to different degrees; perhaps reflecting the importance of the position of the mis-matches in achieving efficient miRNA-directed knockdown of expression. Analysis of complementarity requirements for plant miRNA targeting indicate the position and frequency of mismatches within a given target sequence will influence the level of miRNA-directed repression (Liu et al., 2014). Complementarity at the 3′ end of the target site (5′ end of the miRNA) and the central region are particularly important for efficacy (Mallory et al., 2004; Parizotto et al., 2004; Schwab et al., 2005; Lin et al., 2009). It is likely the frequency and position of the mismatches are responsible for the different levels of miR529b-directed repression exerted upon the different target sequences tested.

The predicted target sequence from rice transcript *OsHKT1;4*, encoding a Na^+^ transporter, produced a relatively modest repression effect. Even though only six replicates were applied to this assay, a simple *t*-test indicated the repression was significant, highlighting the fidelity and applicability of the optimized dual LUC assay in detecting relatively subtle repression effects.

We have extended the application of this assay to assessment of artificial miRNA (amiRNA) and RNAi constructs (data not shown). Artificial microRNA (amiRNA) refers to a technique whereby the mature miRNA sequence of a natural precursor miRNA is replaced with an artificial mature miRNA sequence designed to target an RNA transcript of interest (Ossowski et al., 2008). However artificial microRNA design rules have not yet been perfected, and consequently not all designed amiRNA function efficiently to successfully target the RNA sequence of interest. Recent studies have reported transient expression of amiRNA constructs by agroinfiltration, followed by small RNA Northern blot hybridisation to detect the mature miRNA species (Bhagwat et al., 2013), or co-agroinfiltration of amiRNA and target gene constructs followed by quantitative reverse transcriptase PCR (qRT-PCR) and Western blot analysis to assess efficacy of designed amiRNAs prior to stable transformation (Yu and Pilot, 2014). The optimized assay conditions using the dual LUC reporter system described here provide a faster and scalable alternative to assess amiRNA function.

It is also likely the assay can be extended to assess the ability of other sRNA classes to repress expression of putative target sequences. For example, interactions of sRNAs generated from plant pathogens with complementary plant target sequences could be assessed by comparing the dual LUC ratios generated in infected vs. non-infected tissues. However, assessing target interactions of mobile classes of sRNAs may require an amendment to the protocol where agroinfiltration should occur on just one leaf per plant.

Limitations of the assay include difficulties encountered in assessing miRNA that are endogenous to the host plant. In this paper we applied the optimized assay to assess the interaction of rice miRNA529b with putative rice targets using *N. benthamiana* as the host plant. miR529b is not endogenous to *N. benthamiana*, and therefore we were able to reduce the likelihood of endogenous miRNAs interacting with the target sequences. Using the assay to assess miRNA-target interaction when the miRNA is endogenous to *N. benthamiana* can be problematic, as we have found that reporter-target repression due to abundant endogenous miRNA present in control plants co-agroinfiltrated with a non-functional precursor can be just as severe as in the test plants co-agroinfiltrated with the overexpression miRNA precursor construct (data not shown). This hurdle could be overcome by modifying the control strategy. In such cases, agroinfiltration of the target plasmid could be directly compared to agroinfiltration of a mutated version of the target plasmid to reveal endogenous miRNA-target sequence interaction. However, the possibility of introducing mutations that differentially affect transcript stability cannot be discounted.

There is scope to further improve the assay by, for example, using codon-optimized versions of the firefly LUC and/or Renilla LUC to improve sensitivity (Chou and Moyle, 2014). The more sensitive nanoLUC reporter could also be substituted in place of the Renilla LUC reporter in the dual LUC target cloning plasmid.

This study addresses the challenge of experimentally validating large numbers of computationally predicted transcript sequences that might be targeted by complementary miRNA. The study builds on the use of the dual luciferase assay in combination with agroinfiltration transient expression protocols, by optimizing experimental design and key parameters. The transient basis of the assay allows for a rapid assessment of miRNA action on a target sequence without the need to generate transgenic lines. The quantitative power of the dual LUC assay allows detection of relatively subtle miRNA-target sequence interactions. The optimisation of the assay and careful experimental design allows observation of statistically significant differences in reporter expression from as few as six replicate samples, thereby providing scalability. The assay is versatile and could be applied to observing a number of small RNA-target interactions.

## Author Contributions

RM and PS designed the experiments. RM and LC cloned the constructs. RM, LC, L-SP, GS, and EN performed the dual LUC assays. JD-M performed computational analysis to predict miR529b target sequences. RM and LC analyzed the data. RM drafted the manuscript and all authors edited and approved the final version.

## Conflict of Interest Statement

The authors declare that the research was conducted in the absence of any commercial or financial relationships that could be construed as a potential conflict of interest.

## Footnotes

1 http://www.addgene.org
