# Supplementary material for: An Optimized Transient Dual Luciferase Assay for Quantifying MicroRNA Directed Repression of Targeted Sequences
Source: Front Plant Sci. 2017 Sep 20;8:1631. doi: 10.3389/fpls.2017.01631 (PMC5611435; doi:10.3389/fpls.2017.01631)
Supplement: Supplementary file 1 [file Table_1.DOCX]

List of primers used in the study

| Primer Name | Primer Sequence |
| --- | --- |
| SlActProm_F_FspI | TTTGCGCAGATAATAGTTCGTAAATTTTTGCTCGAG |
| SlActProm_R_SalI | TTTGTCGACTTTTAATCAACTGAAAGTAAAAGCAAGTTAAATC |
| OsSPL14_F | TCGACGAGCTGTGCTCTCTCTCTTCTCTGCA |
| OsSPL14_R | GAGAAGAGAGAGAGCACAGCTCG |
| OsHKT1;4_F | TCGACAAGATGTACTCTTTCTCTTCCCTGCA |
| OsHKT1;4_R | GGGAAGAGAAAGAGTACATCTTG |
| OsFBX292_F | TCGACAGTTATACTCTCTCTCTTCCCTGCA |
| OsFBX292_R | GGGAAGAGAGAGAGTATAACTG |
| OsEthyl_F | TCGACAGCTGTCCTCTCTCTCTCCTCTGCA |
| OsEthyl_R | GAGGAGAGAGAGAGGACAGCTG |
| OsCSLC2_F | TCGACGAGCTTGCTCTCTCTCTTCTCT GCA |
| OsCSLC2_R | GAGAAGAGAGAGAGCAAGCTCG |
| miR529b_PM_F | TCGACGCCCAAAGCTGTACTCTCTCTCTTCTCCATTCTGCA |
| miR529b_PM_R | GAATGGAGAAGAGAGAGAGTACAGCTTTGGGCG |
|  |  |
|  |  |
|  |  |
|  |  |
|  |  |
