# Supplementary material for: An Optimized Transient Dual Luciferase Assay for Quantifying MicroRNA Directed Repression of Targeted Sequences
Source: Front Plant Sci. 2017 Sep 20;8:1631. doi: 10.3389/fpls.2017.01631 (PMC5611435; doi:10.3389/fpls.2017.01631)
Supplement: Supplementary file 2 [file Table_2.DOCX]

psRNAtarget output results for five predicted rice transcript targets of miR529b

| **miR529b** | **Target Acc.** | [**Expectation (E)**](http://plantgrn.noble.org/psRNATarget/?dowhat=Help#maxexpectation) | **Alignment** | **Target Description** | [**Inhibition**](http://plantgrn.noble.org/psRNATarget/?dowhat=Help#validcleavageregion) | [**Multiplicity**](http://plantgrn.noble.org/psRNATarget/?dowhat=Help#multiplicity) |
| --- | --- | --- | --- | --- | --- | --- |
| [agaagagagagaguacagcuu](http://plantgrn.noble.org/psRNATarget/getseq.do?sessionid=1456367042862406&source=srna&seqID=S1) | [LOC_Os08g39890.1](http://plantgrn.noble.org/psRNATarget/getseq.do?sessionid=1456367042862406&source=target&seqID=LOC_Os08g39890.1) | 0.5 | miRNA 21 UUCGACAUGAGAGAGAGAAGA 1  .::::::.:::::::::::::  Target 987 GAGCUGUGCUCUCUCUCUUCU 1007 | cDNA\|OsSPL14 - SBP-box gene family member, expressed | Cleavage | 1 |
| [agaagagagagaguacagcuu](http://plantgrn.noble.org/psRNATarget/getseq.do?sessionid=1456367042862406&source=srna&seqID=S1) | [LOC_Os04g51830.1](http://plantgrn.noble.org/psRNATarget/getseq.do?sessionid=1456367042862406&source=target&seqID=LOC_Os04g51830.1) | 2.5 | miRNA 21 UUCGACAUGAGAGAGAGAAGA 1  ::: ::::::::.:::::::  Target 1646 AAGAUGUACUCUUUCUCUUCC 1666 | cDNA\|OsHKT1;4 - Na+ transporter, expressed | Cleavage | 1 |
| [agaagagagagaguacagcuu](http://plantgrn.noble.org/psRNATarget/getseq.do?sessionid=1456367042862406&source=srna&seqID=S1) | [LOC_Os08g34820.1](http://plantgrn.noble.org/psRNATarget/getseq.do?sessionid=1456367042862406&source=target&seqID=LOC_Os08g34820.1) | 2.5 | miRNA 20 UCGACAUGAGAGAGAGAAGA 1  ::.: ::::::::::::::  Target 482 AGUUAUACUCUCUCUCUUCC 501 | cDNA\|OsFBX292 - F-box domain containing protein, expressed | Cleavage | 1 |
| [agaagagagagaguacagcuu](http://plantgrn.noble.org/psRNATarget/getseq.do?sessionid=1456367042862406&source=srna&seqID=S1) | [LOC_Os05g14010.1](http://plantgrn.noble.org/psRNATarget/getseq.do?sessionid=1456367042862406&source=target&seqID=LOC_Os05g14010.1) | 2.5 | miRNA 20 UCGACAUGAGAGAGAGAAGA 1  :::::: :::::::::: ::  Target 80 AGCUGUCCUCUCUCUCUCCU 99 | cDNA\|OsEthyl - ethylene-responsive protein related, putative, expressed | Cleavage | 1 |
| [agaagagagagaguacagcuu](http://plantgrn.noble.org/psRNATarget/getseq.do?sessionid=1456367042862406&source=srna&seqID=S1) | [LOC_Os09g25900.1](http://plantgrn.noble.org/psRNATarget/getseq.do?sessionid=1456367042862406&source=target&seqID=LOC_Os09g25900.1) | 2.5 | miRNA 21 UUCGACAUGAGAGAGAGAAGA 1  .:::: :.:::::::::::::  Target 1954 GAGCU-UGCUCUCUCUCUUCU 1973 | cDNA\|OsCSLC2 - cellulose synthase-like family C, expressed | Cleavage | 1 |
