# Supplementary material for: An Optimized Transient Dual Luciferase Assay for Quantifying MicroRNA Directed Repression of Targeted Sequences
Source: Front Plant Sci. 2017 Sep 20;8:1631. doi: 10.3389/fpls.2017.01631 (PMC5611435; doi:10.3389/fpls.2017.01631)
Supplement: Supplementary file 3 [file Table_3.DOCX]

Control stem-loop precursor structure used in the study. The non-functional precursor sequence was derived from *Capsicum chlorosis virus*.

GCGGATCCTGGGAATGACAAAGTCAGCTTCACTATAAATATAATGCCAACCTGGAATAGTGGGCGGAGGTTTATGCATATATCCCGGCTTATAGTCTGGGTTGTCCCAACCATCCCAGATTCTAAGAACAGTGTTAAAGCCACTTTAATTGATCAGAACAAGATGACCAAAGCTGACAAAATAGTTATAAGTAGGCAAGCTTCTCTGAAAGACCGAATTCGC
